# Supplementary material for: BUB1 regulates non-homologous end joining pathway to mediate radioresistance in triple-negative breast cancer
Source: J Exp Clin Cancer Res. 2024 Jun 11;43:163. doi: 10.1186/s13046-024-03086-9 (PMC11167950; doi:10.1186/s13046-024-03086-9)

Supplementary Table S1

Mutation status of TNBC cell lines

TNBC Cell Lines	Mutation Status
**SUM159**	BRCA (Wild-type), HRAS (Mutant), PIK3CA (Mutant), TP53 (Mutant)
**MDA-MB-231**	BRAF (Mutant), BRCA (Wild-type), CDKN2A (Mutant), KRAS (Mutant), PIK3CA (Wild-type), TERT (Mutant), TP53 (Mutant)
**MDA-MB-468**	BRCA (Wild-type), PTEN (Mutant), RB1 (Mutant), TP53 (Mutant)
**BT-549**	BRCA (Wild-type), PTEN (Mutant), RB1 (Mutant), TP53 (Mutant)
**T-47D**	BRCA (Wild-type), PIK3CA (Mutant), TP53 (Mutant)

Supplementary Table S2

gRNA sequences for BUB1 knockout

Guide	Sequence	Exon
1	AGCCCACATGCAGAGCTACA	2
2	TTTACTAGAACATTTAATGA	3

Primers for PCR amplification of BUB1-edited section

Primer	Sequence
Forward	TGACATTGGGGTTCCGTGAG
Reverse	TCACTAGTGGCACAGAAGTCT

Primer for Sanger sequencing

Primer	Sequence
Forward	TGACATTGGGGTTCCGTGAG

Supplementary Table S3

List of antibodies used for Western Blotting/Immunohistochemical /Immunofluorescence studies

Antibody	Company	Catalog Number	Dilutions Used
Anti-rabbit IgG, HRP-linked Secondary	Cell Signaling	7074s	1:10,000
Anti-mouse IgG, HRP-linked Secondary	Cell Signaling	7076s	1:10,000
Anti-Ku70 Rabbit mAb	Cell Signaling	4104S	1:1000
Anti-Ku80 Rabbit mAb	Cell Signaling	2180s	1:1000
DNA-PKcs Phospho antibody (S2056) (E9J4G) Rabbit mAb	Cell Signaling	68716	1:1000
DNA-PKcs Antibody Rabbit mAb	Cell Signaling	4602s	1:1000
Phospho-ATM (Ser1981) Rabbit mAb	Cell Signaling	13050S	1:1000
ATM (D2E2) Rabbit mAb	Cell Signaling	2873S	1:1000
Phospho-KAP-1 (Ser824) Rabbit mAb	Cell Signaling	4127S	1:1000
KAP-1 Antibody Rabbit mAb	Cell Signaling	4123S	1:1000
Lamin B1 (D4Q4Z) Rabbit mAb	Cell Signaling	12586S	1:1000
Recombinant anti-BUB1 Rabbit mAb	Abcam	ab195268	1:1000
B-Actin Mouse mAb (HRP Conjugate)	Cell Signaling	12262	1:30000
Ki67 antigen (Dako Omnis) Clone MIB-1 Mouse monoclonal anti-human antibody	Agilent	GA626	Ready-to-use
Anti-phospho-Histone H2A.X (Ser139)	Millipore	05-636-I	1:2000
Goat anti-Mouse, Alexa Fluor 488	Thermo Fisher Scientific	A-11001	1:2000

Supplementary Table S4

Primer sequences used in quantitative PCR (qPCR) analysis

Target Gene	Primer	Sequence (5'-3')	Tm⁰
GAPDH	Forward	TCGGAGTCAACGGATTTG	62.4
	Reverse	CAACAATATCCACTTTACCAGAG	59.1
BUB1	Forward	GATCGATTACTTTGGGGTTG	60.8
	Reverse	AAAAAGACCTTCAGGCTTAC	56.6
BAX	Forward	TCTGAGCAGATCATGAAGAC	58
	Reverse	TCCATGTTACTGTCCAGTTC	57.5
BCL2	Forward	GATTGTGGCCTTCTTTGAG	59.8
	Reverse	GTTCCACAAAGGCATCC	59
PCNA	Forward	CTGTGTAGTAAAGATGCCTTC	55.6
	Reverse	TCTCTATGGTAACAGCTTCC	56
PRKDC	Forward	GATCTGAAGAGATATGCTGTG	56.4
	Reverse	GTTTCAGAAAGGATTCCAGG	60.1
H2AFX	Forward	AATCCAAGCACCTAGATACC	57.2
	Reverse	CAGAATTCCAGTTCAGAAGC	59
XRCC5	Forward	CAGTGAGAGTCTGAGAAAAC	53.8
	Reverse	TAGGCTGCAATCCTTATAGAC	57.6
XRCC6	Forward	AAGAAGAGTTGGATGACCAG	58.3
	Reverse	GTCACTTCTGTATGTGAAGC	54.6
CASP3	Forward	AAAGCACTGGAATGACATC	57.6
	Reverse	CGCATCAATTCCACAATTTC	62.8
CASP9	Forward	CTCTACTTTCCCAGGTTTTG	57.9
	Reverse	TTTCACCGAAACAGCATTAG	60.3

Supplementary Fig. S5

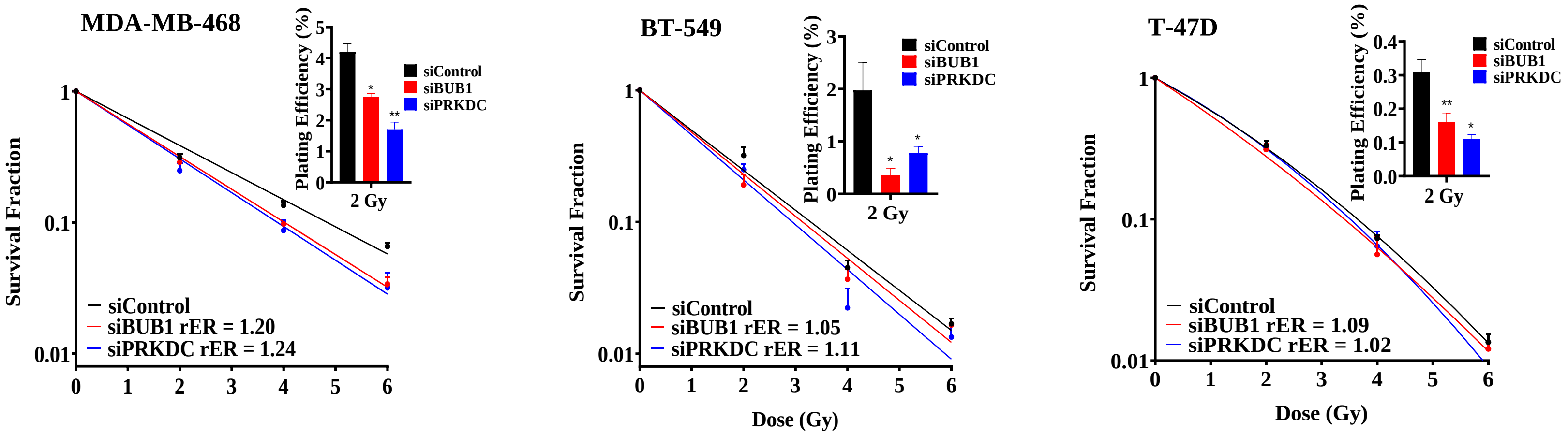

Supplementary Fig. S6

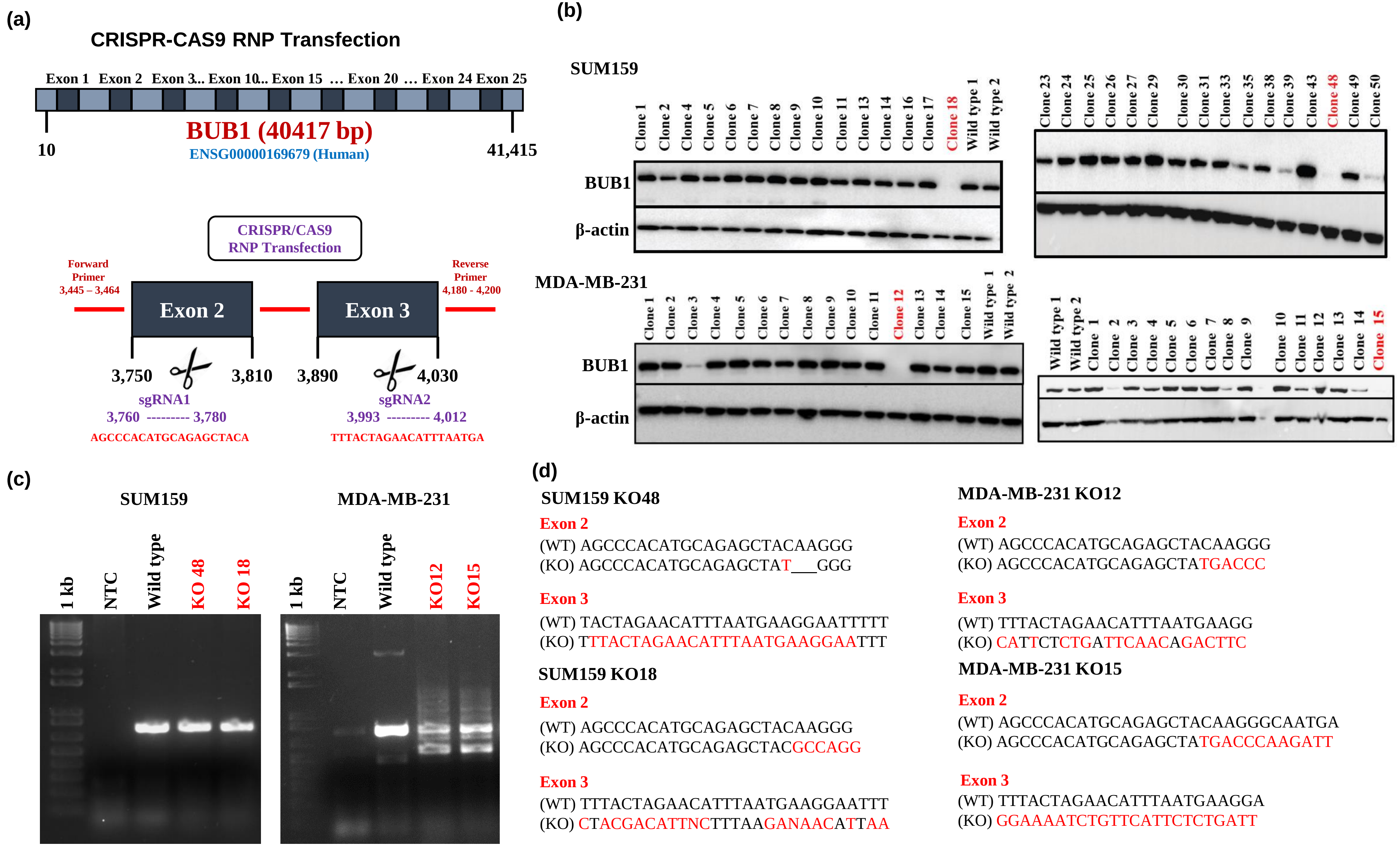

(e)

SUM159 KO48

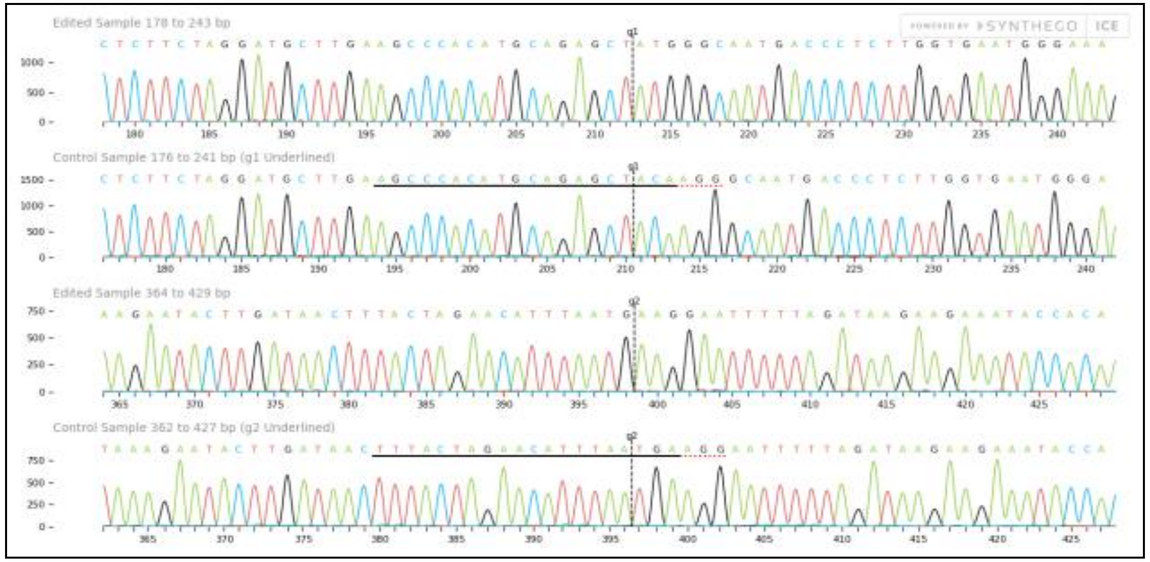

MDA-MB-231 KO12

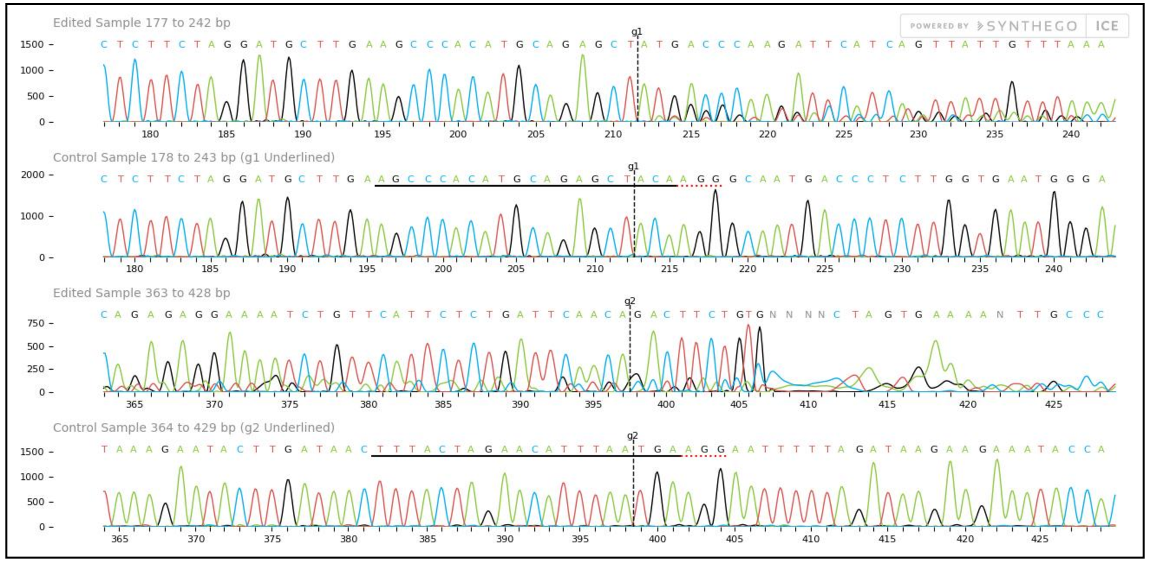

SUM159 KO18

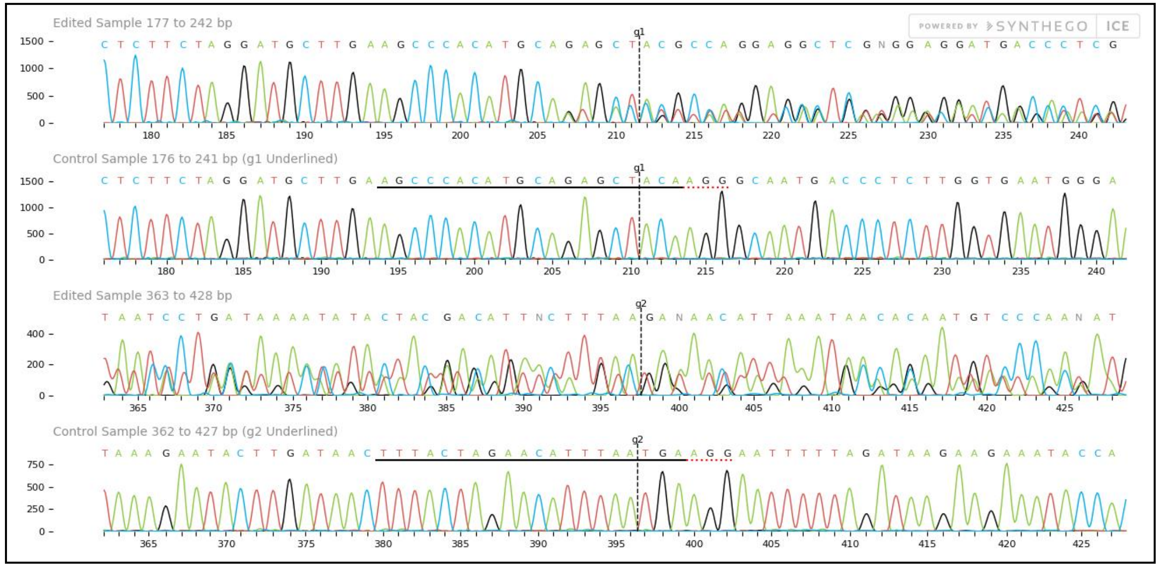

MDA-MB-231 KO15

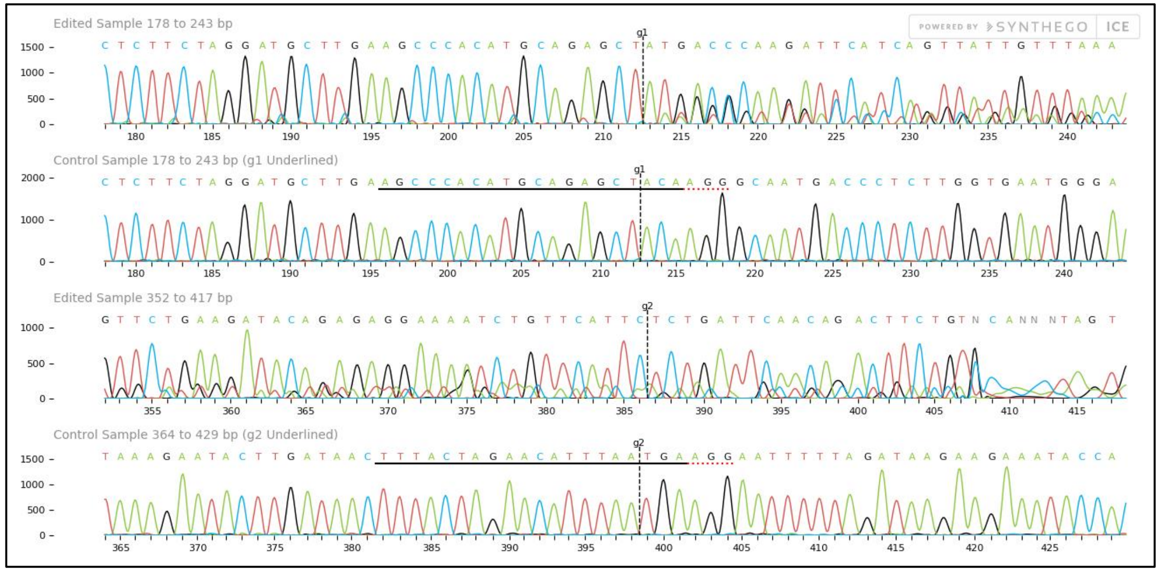

Supplementary Fig. S7

(a)

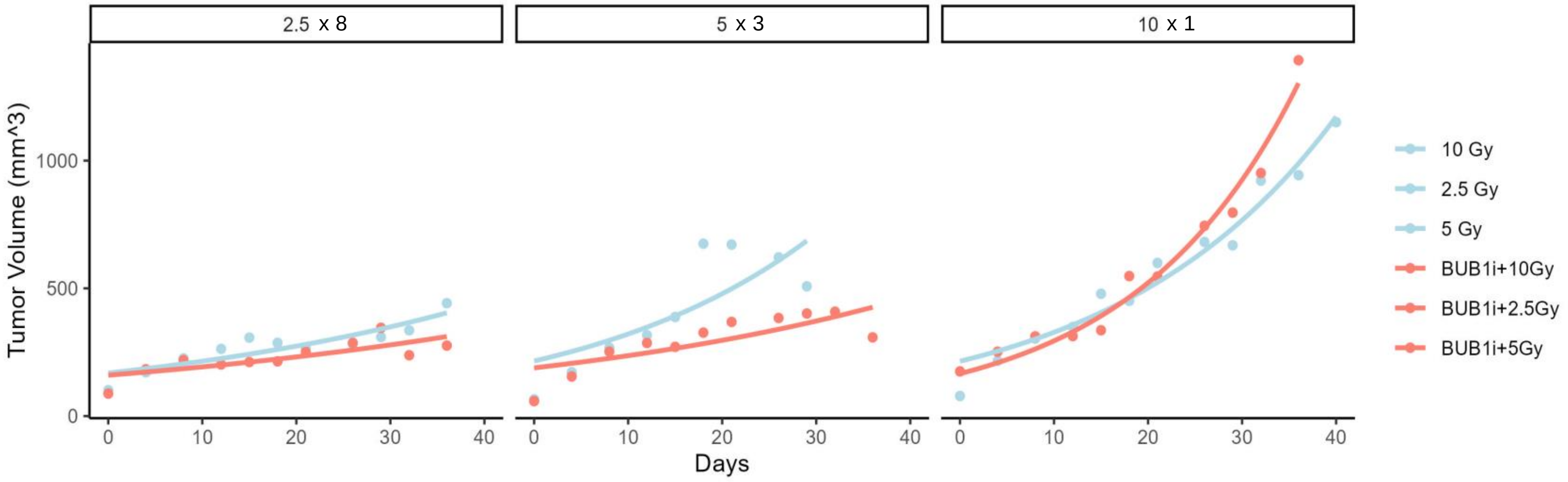

(b)

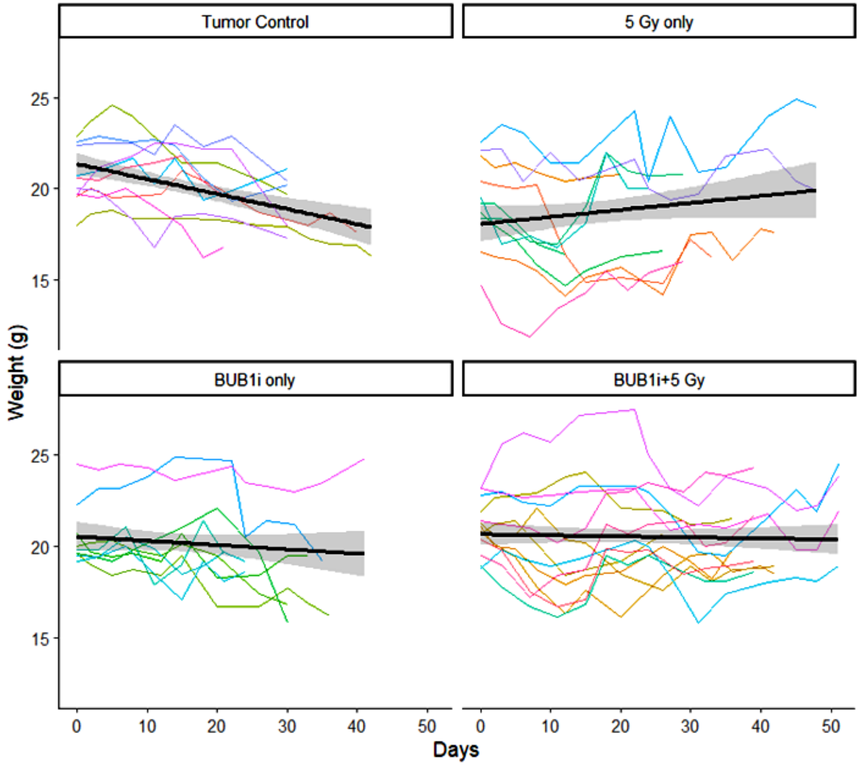

Supplementary Fig. S8

(a)

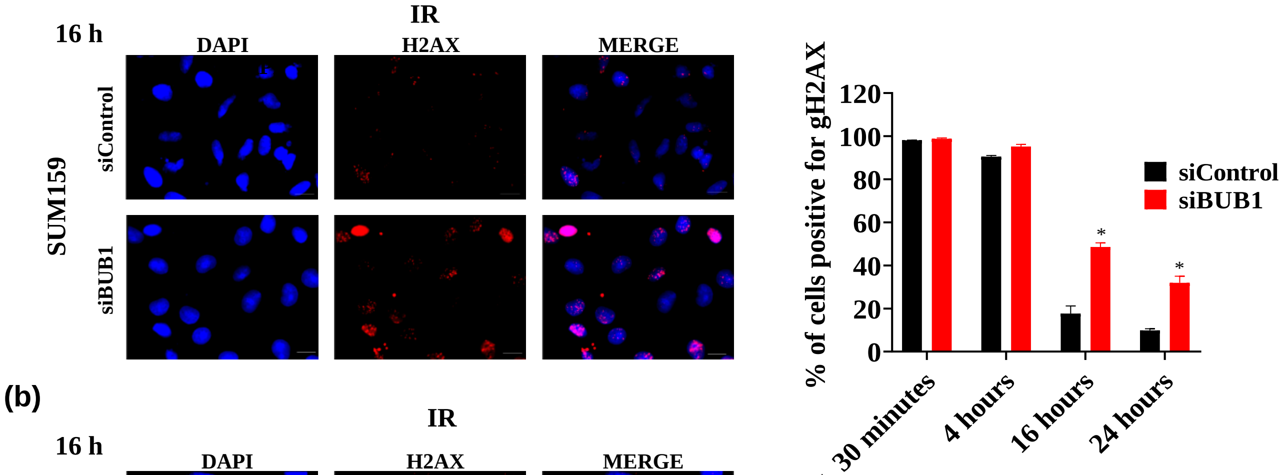

(b)

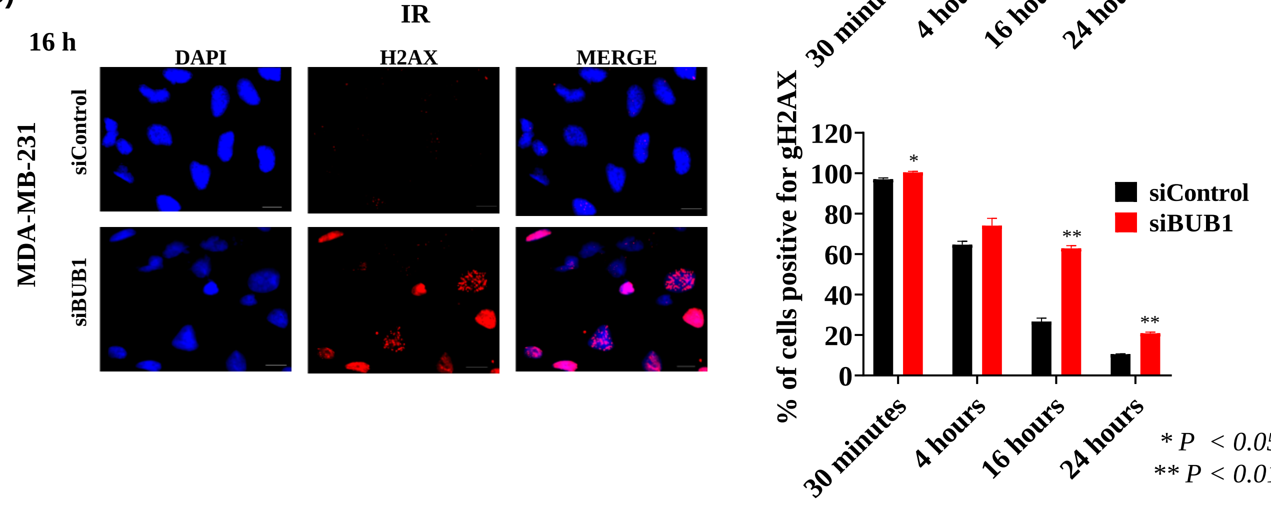

* $P < 0.05$
** $P < 0.01$

Supplementary Fig. S9

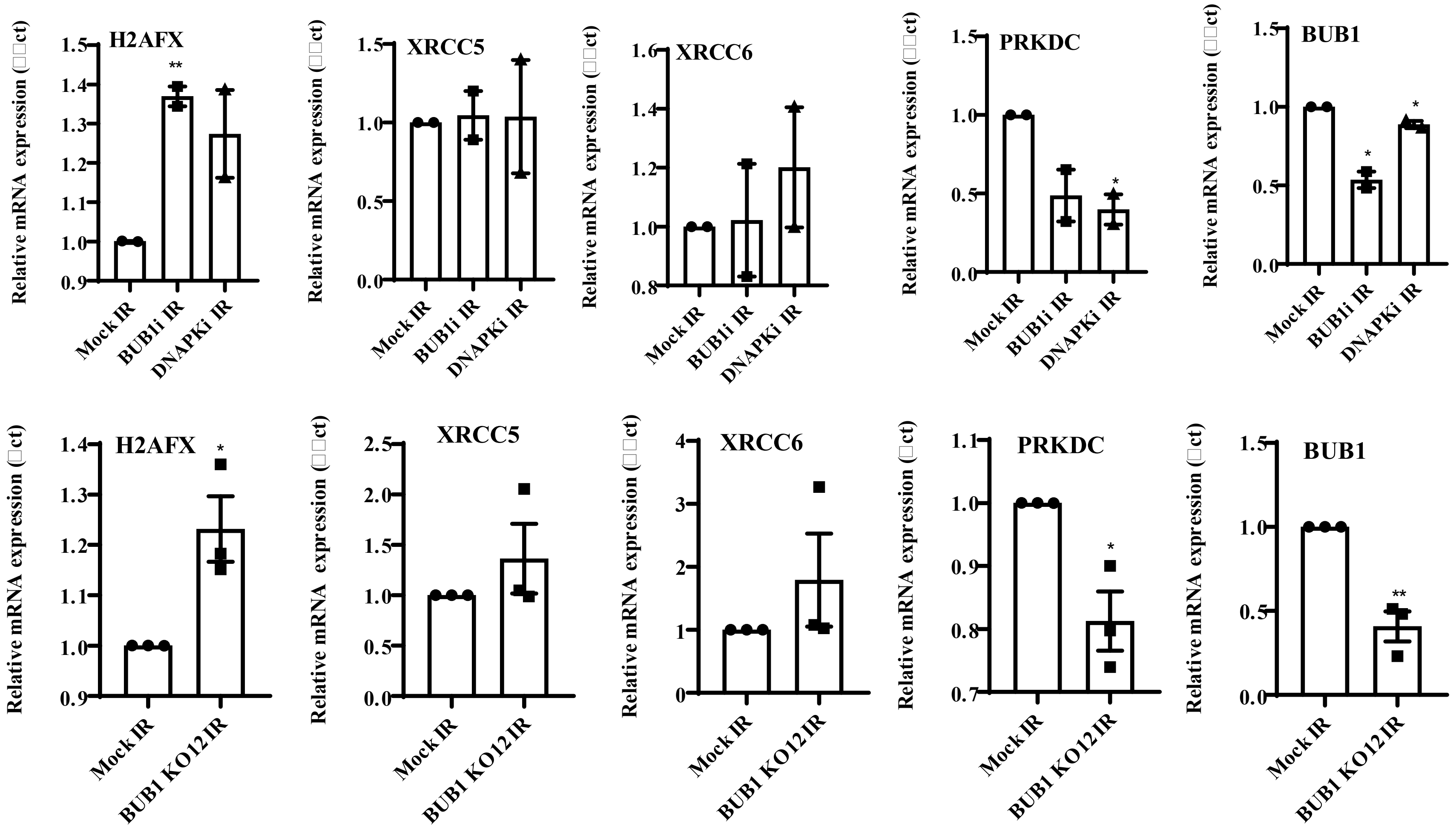

Supplementary Fig. S10

(a)

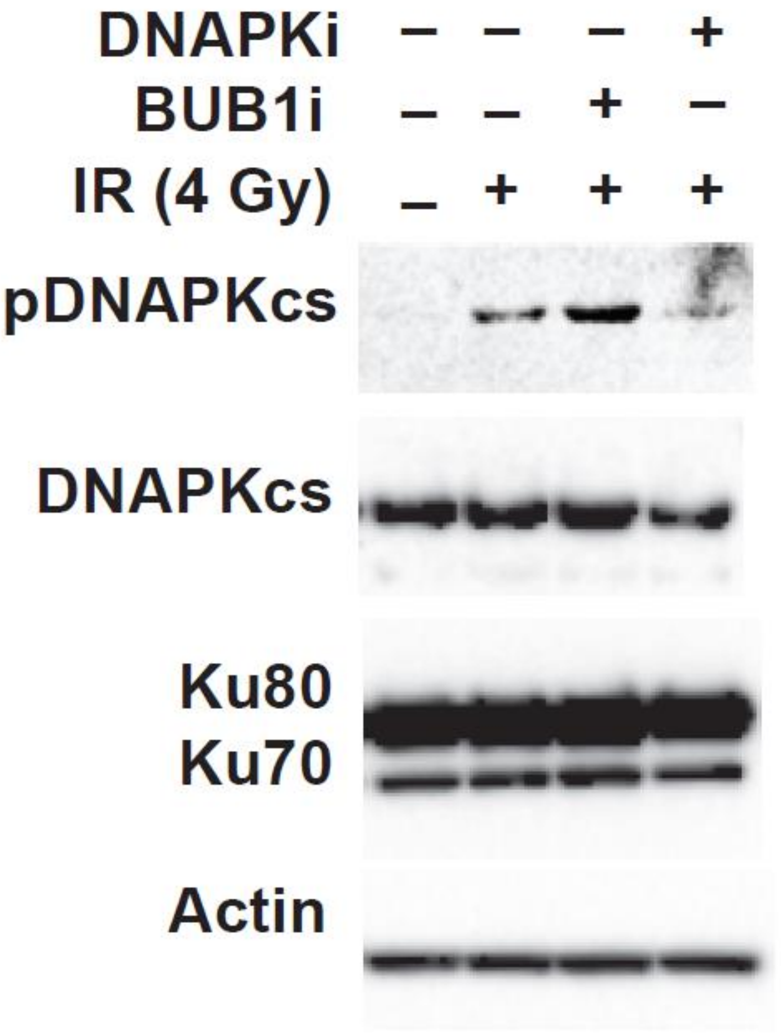

(b)

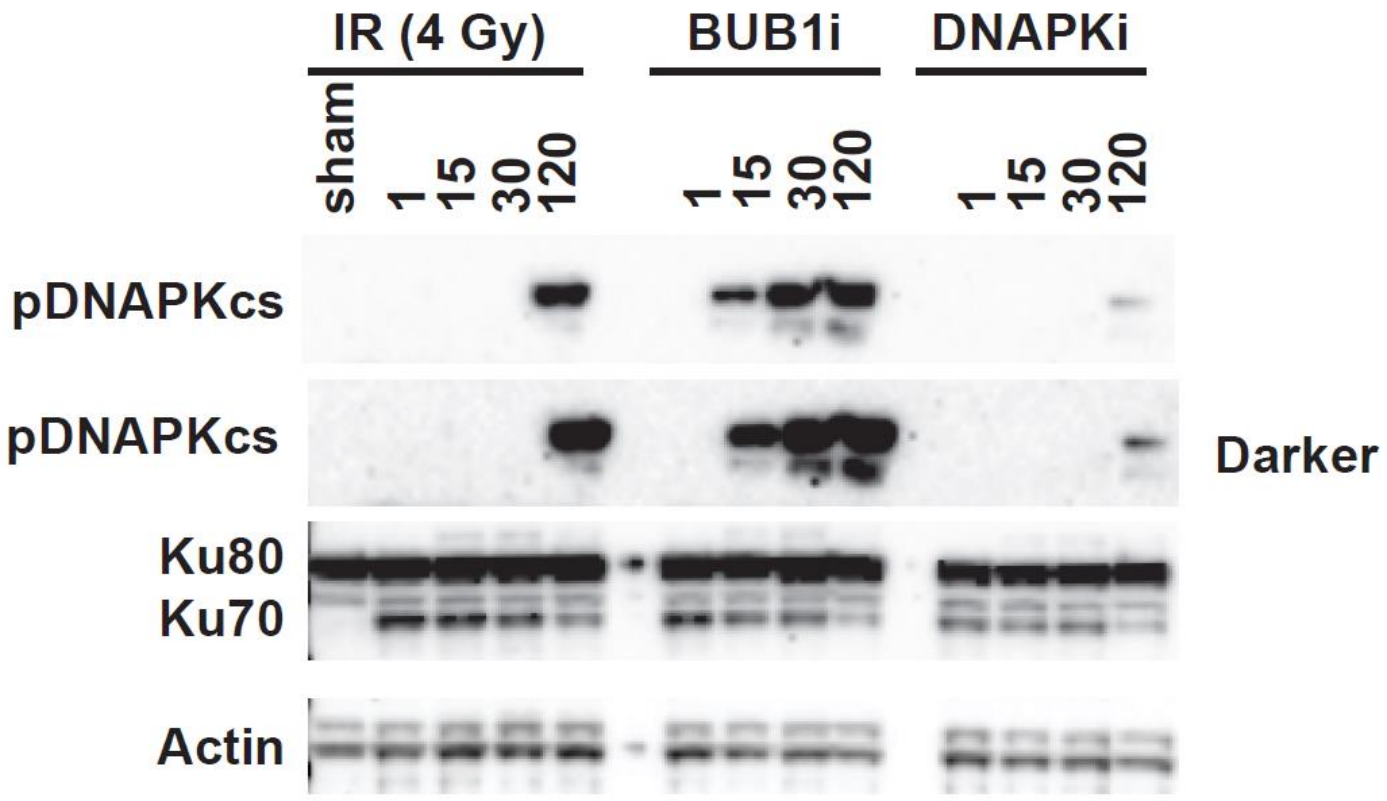

Supplementary Fig. S11

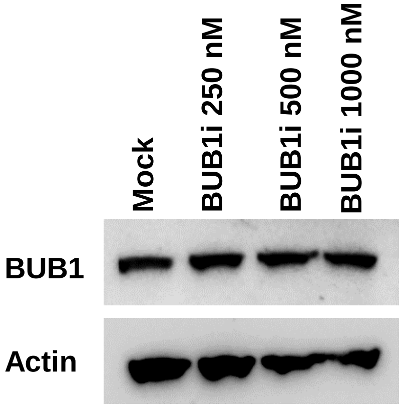

Supplementary Fig. S12

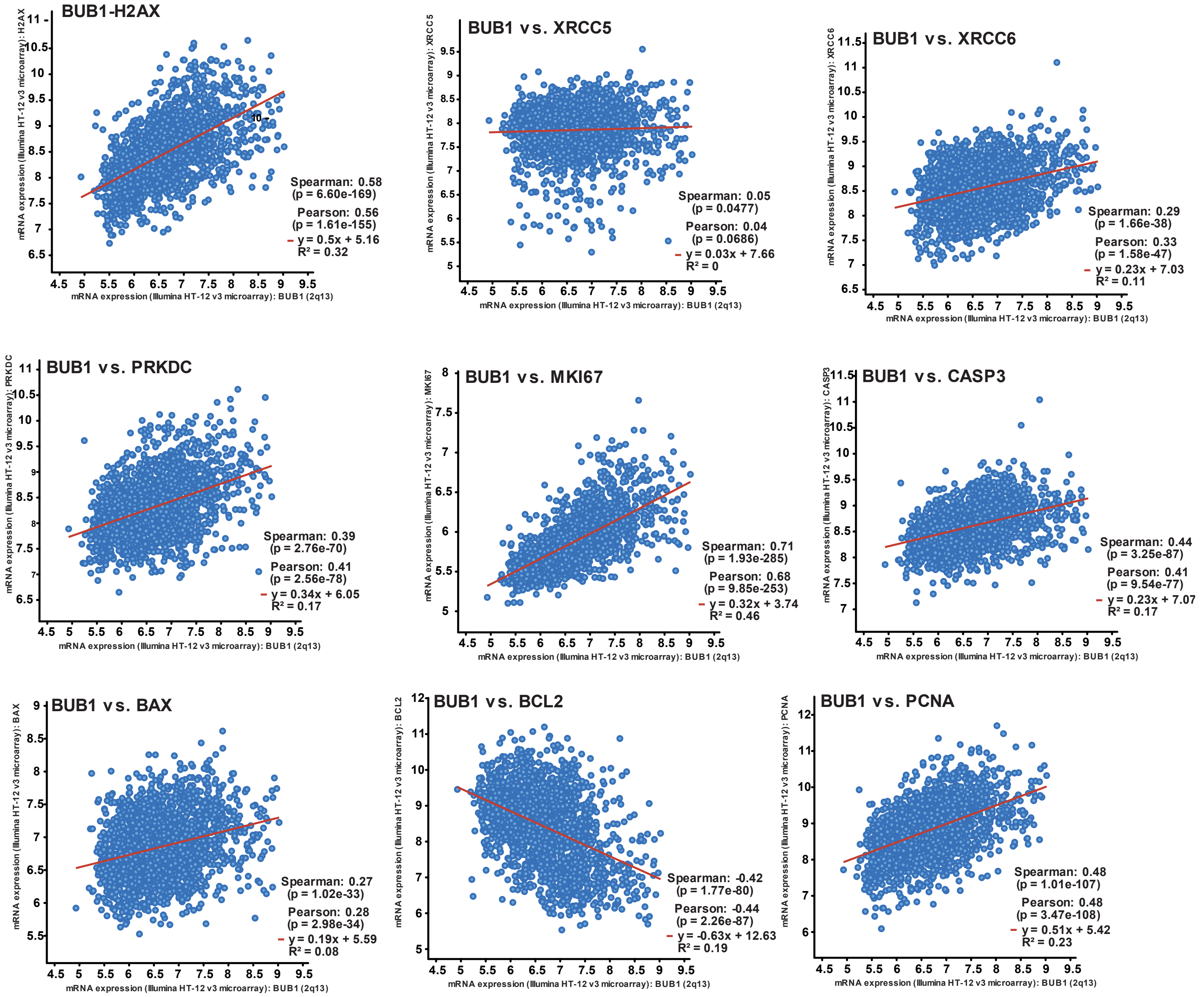

Supplementary Fig. S13

(a)

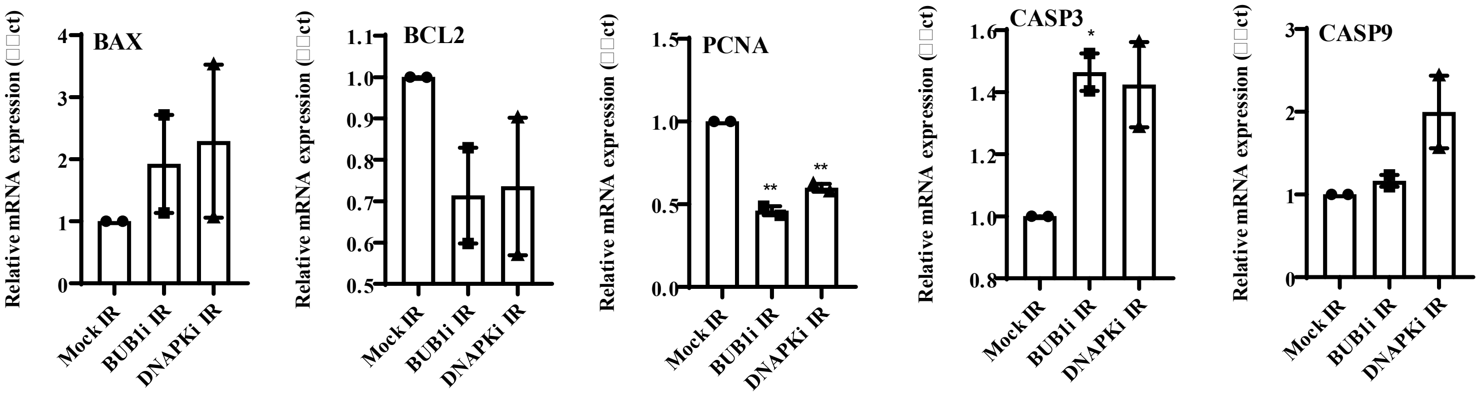

(b)

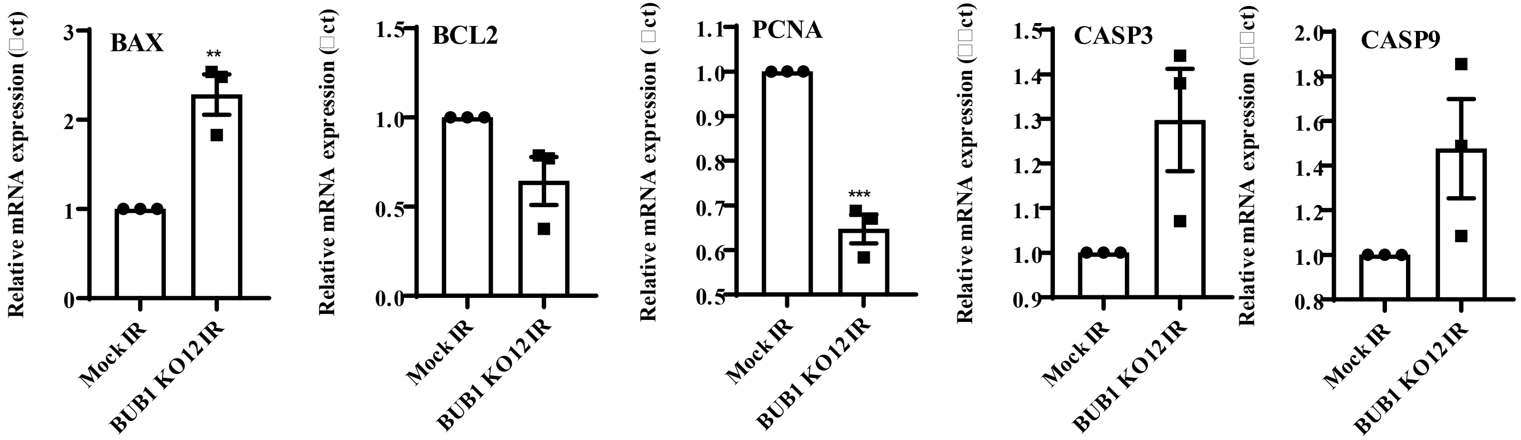

Supplement: Supplementary file 1 — Additional file 1: Table S1. List of mutated genes in the TNBC cell lines. Table S2. Guide RNA (gRNA) sequences used to knock out BUB1, primer sequences for PCR amplification of BUB1-edited section, and primer sequence for Sanger sequencing. Table S3. List of antibodies used for Western Blotting/Immunohistochemical /Immunofluorescence studies. Table S4. Primer sequences used in quantitative PCR (qRT-PCR) analysis. Fig. S5. Clonogenic assays using BUB1 siRNA and RT in (A) MDA-MB-468, (B) BT-549, (C) T-47D cell lines. PRKDC siRNA is used as a positive control. Fig. S6. (A) CRISPR-CAS9 RNP transfection method was utilized to knock out BUB1 (B) BUB1 knockouts were confirmed through Immunoblotting in SUM159, and MDA-MB-231 cell lines followed by (C) PCR amplification and (D) Sanger Sequencing to further validate the BUB1 KO’s (E) Sanger Sequencing chromatograms of SUM159 BUB1 KO #48 and #18, and MDA-MB-231 KO #12 and #15. Fig. S7. (A) Initial radiation dose-response studies in SUM159 tumor xenograft in CB17 SCID mice. SUM159 xenograft mammary fat pad tumors were conformally irradiated at 2.5 GyX8, 5 Gy X3 or 10 GyX1 by SARRP (light blue curves). Additionally, mice were treated with a BUB1 inhibitor (25 mg/kg, orally, twice daily, 5 days/week for 4 weeks) along with radiation (red curves). (B) A spaghetti plot for animal body weight change during the treatment. Fig. S8. Immunofluorescence studies using BUB1 siRNA and RT (16 h time point) in (a) SUM159 and (b) MDA-MB-231 cell lines. Fig. S9. qRT-PCR of NHEJ pathway related genes in MDA-MB-231 cell line with BUB1i (top panel) and BUB1 CRISPR-KO #12. Fig. S10. Effect of BUB1 inhibition with IR on DNAPKcs phosphorylation using Immunoblotting in (A) MDA-MB-468 cell line, and (B) shown at different time points up to 2 h. Fig. S11. The effect of BUB1 inhibitor (BAY1816032) on BUB1 protein levels in normal mammary epithelial cell line MCF 10A. The cells were treated for 1 hour with the same doses of BUB1i that were used for the c [file 13046_2024_3086_MOESM1_ESM.pdf]
